# Role of surgical simulation on self-reported confidence level on cardiothoracic surgical trainees

**DOI:** 10.1186/s13019-024-02647-5

**Published:** 2024-05-18

**Authors:** Len En Yean, Shahrul Amry bin Hashim

**Affiliations:** Cardiothoracic Unit, Department of Surgery, Universiti Malaya Medical Centre, Jalan Profesor Diraja Ungku Aziz, Kuala Lumpur, 59100 Malaysia

**Keywords:** Simulation, Minimally invasive cardiac surgery, Confidence level

## Abstract

**Background:**

Simulated self-practice using simulation models could improve fine motor skills and self confidence in surgical trainees.

**Aims:**

The purpose of this study is to evaluate on self-reported confidence level in cardiothoracic surgical trainees by using surgical simulation models.

**Methods:**

We conducted a cross-sectional study on all surgeons (*n*=10) involved in MIS simulation training. All surgeons are required to perform on three minimally invasive surgery (MIS) procedures (Mitral Valve Repair, Mitral Valve Replacement and Aortic Valve Replacement). A questionnaire was designed based on two existing scales related to self-confidence, the surgical self-efficacy scale [SSES] and the perceived competency scale [PCS]. We assessed their self-confidence (before and after training) in the use of simulation in MIS procedures using rating scales 1-5. The mean score was calculated for each domain and used as the predictor variable. We also developed six questions (PCS) using Objective Structured Assessment of Technical Skills (OSAT) related to each domain and asked participants how confident they were after performing each MICS procedure.

**Results:**

The mean score was 4.7 for all assessed domains, except "knowledge" (3.8). Surgeons who had performed one or more MIS procedures had higher scores (*P*<0.05). There was no correlation between the number of MIS procedures performed and self-confidence scores.

**Conclusions:**

The results indicate that the cardiac surgery training based on MIS simulation improves trainees and consultants in terms of the level of self-confidence. Although surgeons generally have high levels of self-confidence after simulation training in MIS cardiac procedures, there is still room for improvement with respect to technical skills related to the procedure itself and its results.

## Introduction

Self-confidence is a state of mind marked by an individual's belief in his or her ability to succeed at a task or tasks. It is generally seen as a positive trait, although too much self-confidence can lead to carelessness and arrogance, while too little can lead to insecurity and self-doubt. A lack of self-confidence may be due to an individual's poor personal performance relative to others, such as in sports or work situations.

Self-confidence is an important attribute for surgeons and healthcare professionals. It is an essential aspect of surgical performance and a reliable and valid self-confidence scale can help improve training and assessment [1]. A cross-sectional study was conducted among residents of general surgery in the United States, revealing that 27.5% were not confident when performing procedures independently [2]. To be successful in clinical practise, it is crucial to possess critical thinking skills and self-confidence [3].

Minimally invasive surgical (MIS) techniques have become the standard of surgical care in the 21^st^ century and are highly suitable for simulation-based training due to their inherent nature compared to open surgery [4]. A simulator allows trainees to improve their skills and performance with a particular technique or new instrument during training [5].

Objective Structured Assessment of Technical Skills (OSATS) has been used to assess surgeon technical skills in laparoscopic surgery [6]. OSATS has been used locally as a standard for evaluating surgical training programmes and as an assessment tool for individual surgeons seeking certification. It provides data on how well surgeons use technical skills in patient management, but does not provide information on how confident they are about performing these skills or how often they use them in their daily practise. In this study, we sought to evaluate a self-confidence scale by using OSATS for participants.

## Methods

**Fig. 1:**
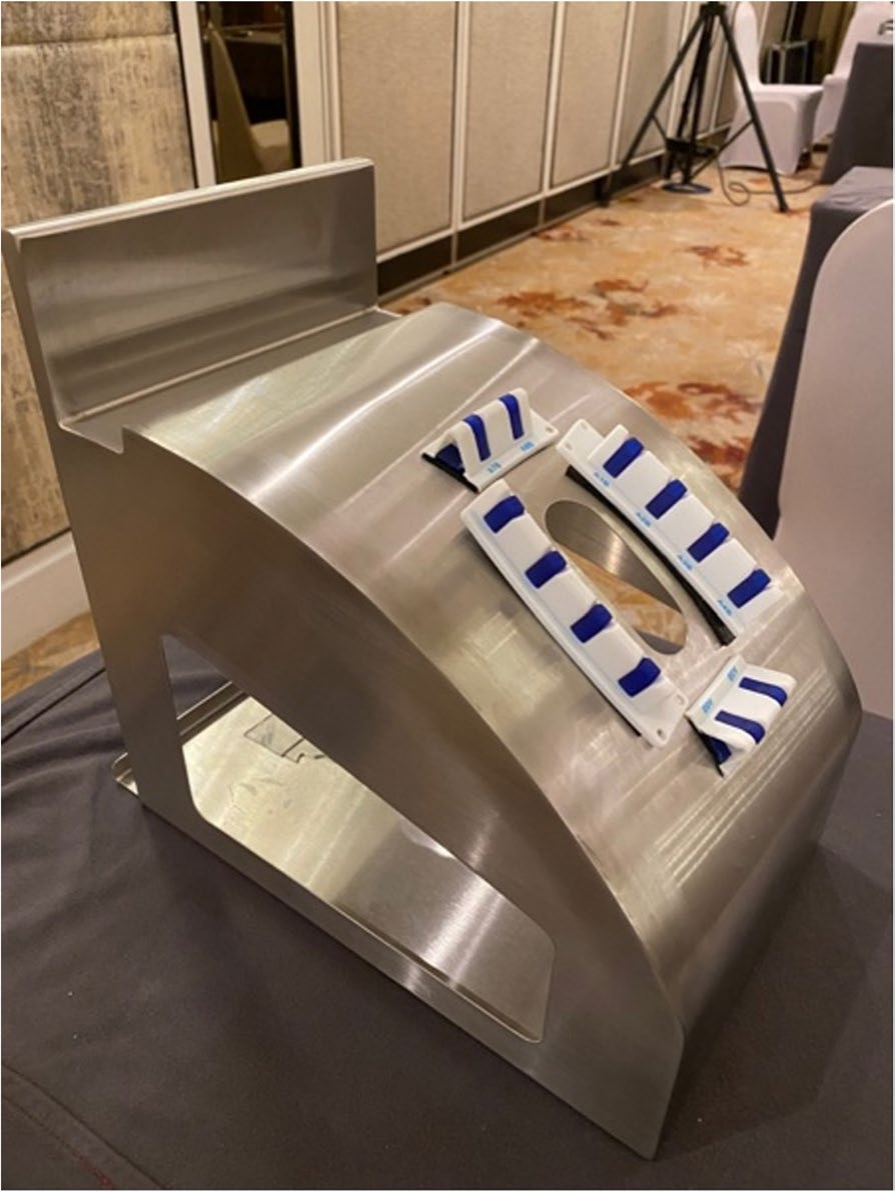
Surgical simulation tools on minimally invasive cardiac surgery training

**Fig. 2:**
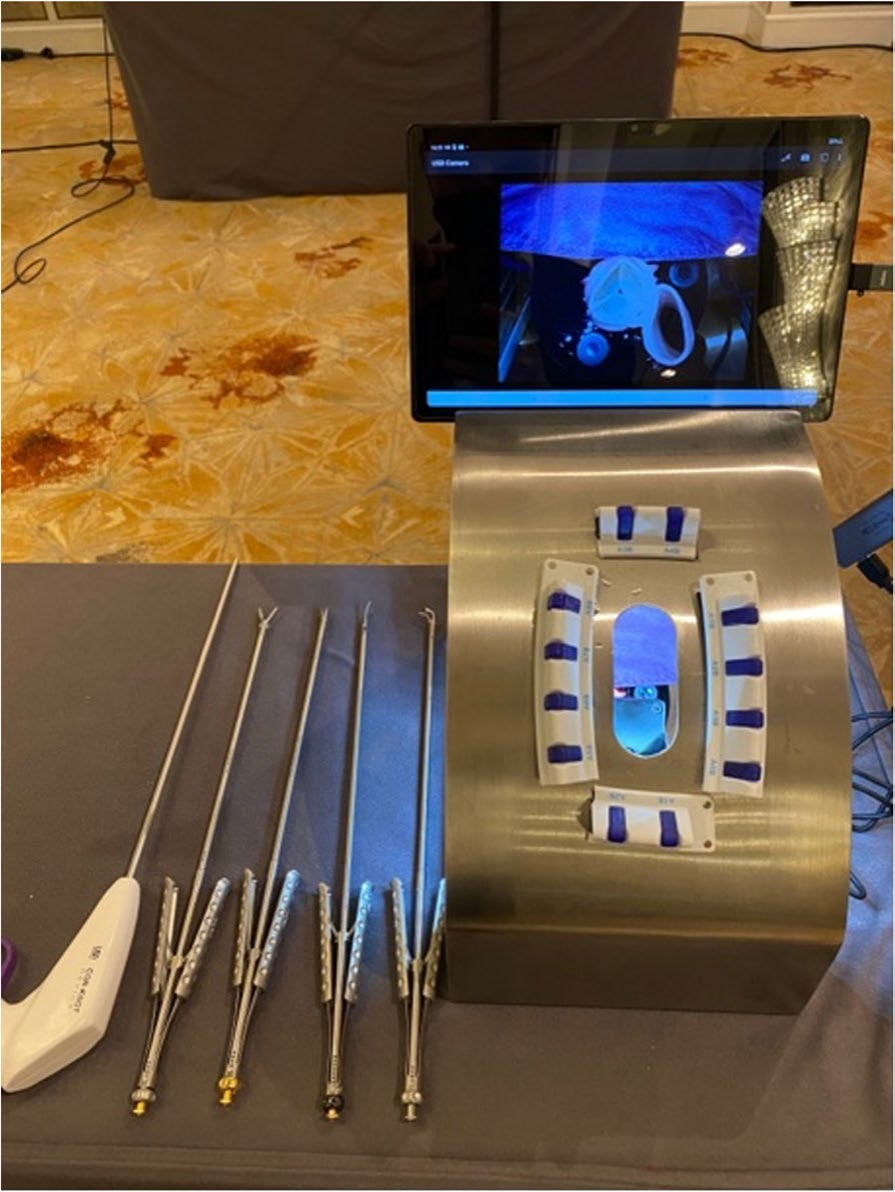
Surgical simulation tools on minimally invasive cardiac surgery training

In this study, we aimed to evaluate a self-confidence scale for surgical trainees and consultants using a self-designed simulation tools in minimally invasive cardiac surgery (MIS). Participants consist of five final year surgical trainees and five consultants which had at least three years of working experience at a consultant level. They all were from different institution and had comparable training and skills levels. We use a simulation tools (as shown in Figs. 1 & 2) in minimally invasive surgery (MIS) for cardiac surgery. All surgeons are required to perform on three MIS procedures (Mitral Valve Repair, Mitral Valve Replacement and Aortic Valve Replacement). We hypothesised that the instrument would be able to distinguish between the two groups of surgeons (trainees and consultants) and that the level of confidence would be higher between consultants than among trainees. Data were collected using survey questionnaires and analysed using descriptive statistics, t tests, and ANOVA.

**Fig. 3:**
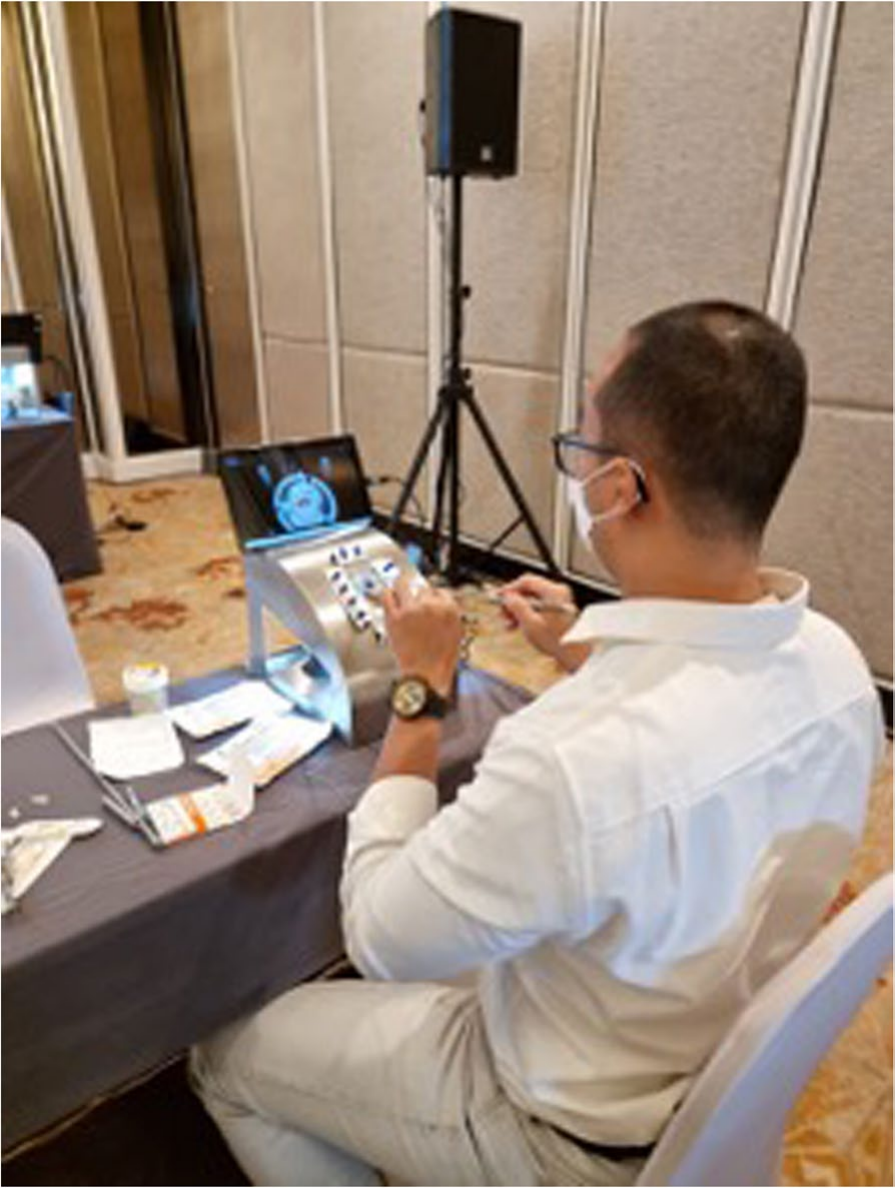
Training in the simulator

**Fig. 4:**
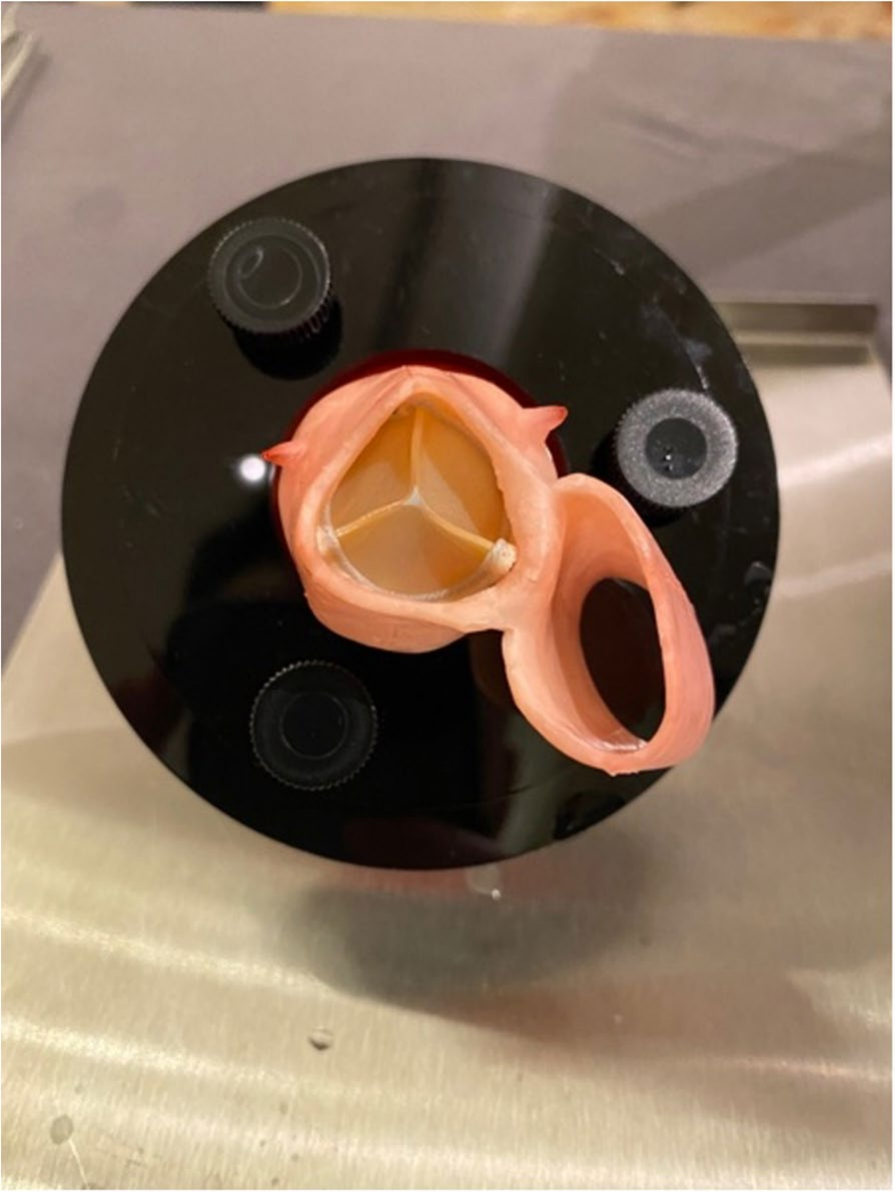
Aortic valve replacement model

A questionnaire was designed based on two existing scales related to the surgical self-efficacy scale [SSES] of self-confidence and the perceived competence scale [PCS]). We developed six questions related to each domain in PCS and asked participants how satisfied and confident they were after performing each MICS procedure (as shown in Figs. 3 and 4).

**Fig. 5:**
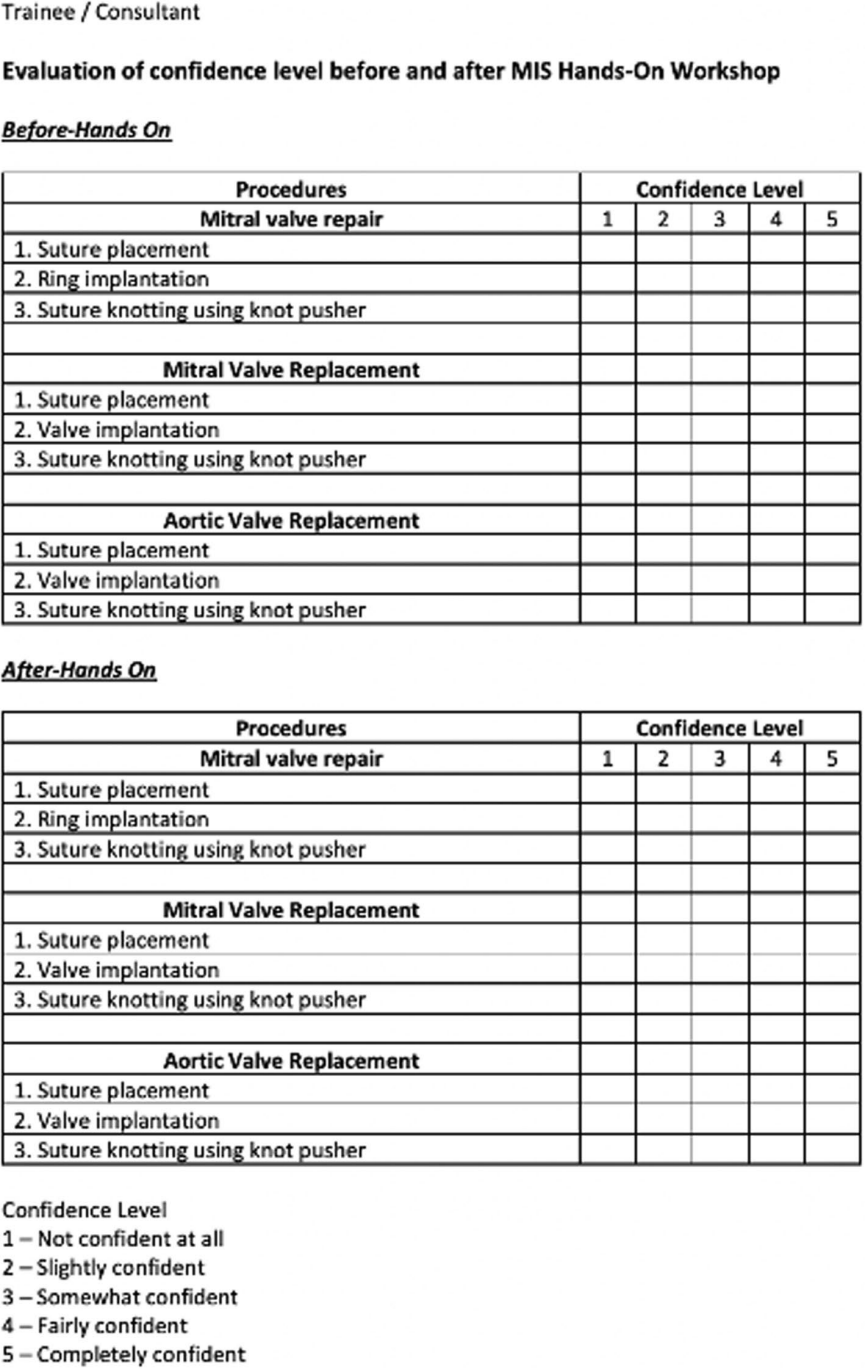
Surgical Self-Efficacy Scale (SSES) Questionnaire for Confidence Level

To evaluate the effect of a hands-on workshop on the level of self-confidence of surgical trainees and consultants in the use of simulation tools in minimally invasive cardiac surgery, we designed a prospective observational study with two groups: group A (*n* = 5) consisting of surgical trainees and group B (*n* = 5) consisting of consultants. All participants completed the SSES questionnaire before and after a hands-on workshop on MIS. The study population included all surgical trainees and consultants working in the cardiothoracic surgical field.

The SSES questionnaire is designed to assess their self-confidence (before and after training) in the use of simulation tools in MIS cardiac procedures through rating scales 1-5. From 1 not being confident at all to 5 being completely confident. Each procedure is divided into suture placement, ring/valve implantation, and suture knotting using a knot pusher (as shown in Fig. 5). Data were assessed by comparing mean scores obtained by the two groups (trainees vs consultants), depending on whether the data was normally distributed.

**Fig. 6:**
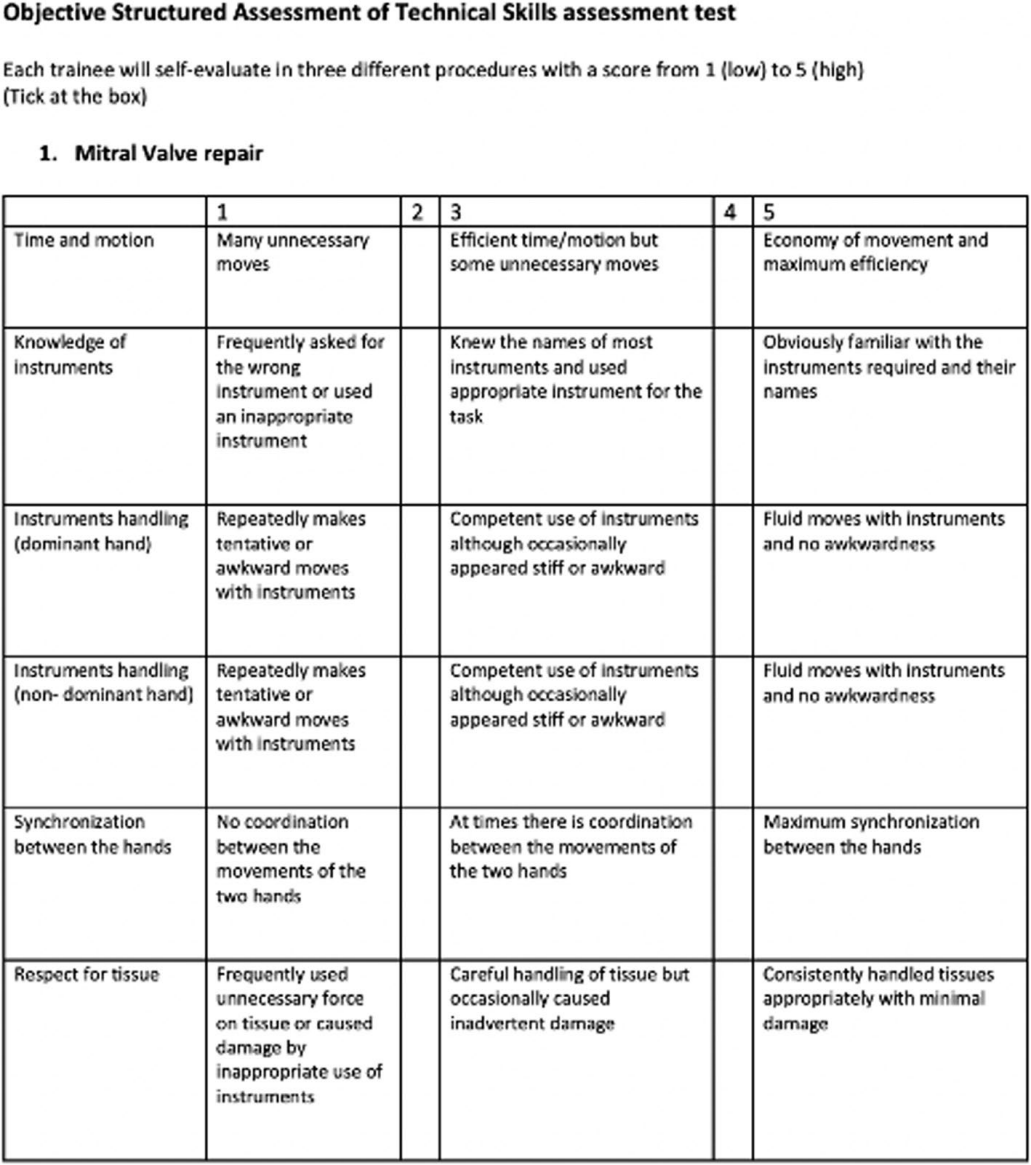
Objective Structured Assessment of Technical Skills (OSAT) form for perceived competency scale (PCS) assessment

The PCS questionnaire includes six items on surgeons' confidence in MIS simulator training, which include time and motion, knowledge of instruments, instrument handling using dominant and nondominant hand, synchronisation between hands, and respect for tissue. Each item had 5 possible responses, from the rating scales 1-5, based on their satisfaction and confidence (as shown in Fig. 6). Three separate questionnaires for 3 different MIS procedures training (Mitral valve repair, Mitral Valve replacement and Aortic valve replacement) were filled in by the participants after their training, The data was assessed by comparing mean scores for all domains.

## Results

For the surgical self-efficacy scale [SSES], the results showed that there was a significant difference between the two groups in terms of their level of confidence after MIS training (*p* < 0.05) in training for the three procedures (mitral valve repair, mitral valve replacement, aortic valve replacement). Two groups had a significant increase in confidence level in terms of suture placement, ring / valve implantation, and suture knotting using a knot pusher. For all the three procedures, the mean score was 2.6 before the training and ranging from 4.3-4.5 after the training.

The results showed that the before and after training scores were significantly higher in both groups (surgical trainees and consultants) after the hands-on workshop than before (*p*<0.05). There were no significant differences between groups A and B (*p*>0.05)

In terms of the perceived competence scale [PCS] using Objective Structured Assessment of Technical Skills (OSATS), the mean score for the 3 MIS procedures was lower than 4 (scale range 0–5) for all evaluated domains (time and motion, handling of instruments using the dominant and non-dominant hand, synchronisation between hands, respect for tissue) except for "knowledge of instruments" (> 4). The mean scores of all domains except knowledge ranged from 3.5-3.9.

## Discussion

The use of simulation in minimally invasive cardiac surgery (MICS) has been rising over the last decade. In view of increasing patient demand for less invasive surgery, it is important that cardiovascular surgeons remain familiar with the most widely used approaches to surgery [7]. Training in cardiac surgery using simulation has demonstrated a substantial influence on the clinical knowledge, surgical skills (including accuracy, timing, and dexterity), and confidence of trainees in addressing cardiac surgical scenarios, in regardless both junior and senior levels [8].

Studies have shown that surgeons who have undergone additional simulation training improve significantly in terms of surgical skill [9]. However, there are still many concerns regarding effectiveness and cost-effectiveness; Simulators have proven to be effective in cost-effectively teaching complex laparoscopic skills with the current surgical training, as discussed in the paper [10].

Simulation based training program has been proven to improve not only performance skills but also self confidence among trainees [11]. The self-confidence scale can be used to identify trainees who may benefit from additional training and support and to assess the effectiveness of training programmes [1].

In this study, we developed a self-confidence scale for surgeons to evaluate their confidence in using simulation and compared surgical trainees and consultants. The results showed that both groups had moderate self-confidence in using simulation to develop minimally invasive cardiac surgery skills, but this was significantly higher among consultants compared to trainees. However, there were no differences between the two groups in their perception of the importance of using simulation in teaching surgical skills. This is consistent with previous studies that have found that surgeons believe that the use of technology can improve skill acquisition and lead to better outcomes [12].

Although this study was conducted in a single institution, similar findings may be obtained in other institutions, as many hospitals use simulators to teach skills related to minimally invasive cardiac surgery.

However, this study is only conducted in a small sample size. Therefore, it is recommended that further studies with larger samples be conducted to establish if there are differences between these two groups with respect to their level of confidence in the training of MIS simulation tools for cardiac surgery.

To date, there has been no published work exploring whether surgeons’ perceptions about the importance of using simulators differ according to speciality or experience levels.

## Conclusions

This study provides a new way to evaluate surgical self-confidence using an easy questionnaire with good psychometric properties. The self-designed MIS simulation tools also can be used as an evaluation tool during training courses or when evaluating surgical trainees or consultants before starting a procedure to assess their self-confidence before starting the procedure. Our study has shown that this scale can be used effectively to evaluate surgical trainees and consultants in the use of simulation for minimally invasive cardiac surgery. The results indicate that a single adjective rating can help measure tools for training and self-improvement in surgical skills.

## References

[1] Geoffrion R (2013). Validating a self-confidence scale for surgical trainees. J Obstet Gynaecol Can.

[2] Yeo H (2009). Attitudes, training experiences, and professional expectations of US general surgery residents: a national survey. JAMA.

[3] Alamrani MH (2018). Comparing the effects of simulation-based and traditional teaching methods on the critical thinking abilities and self-confidence of nursing students. J Nurs Res.

[4] Schreuder HW (2011). Implementation of simulation in surgical practice: minimally invasive surgery has taken the lead: the Dutch experience. Med Teach.

[5] Derossis AM (1998). The effect of practice on performance in a laparoscopic simulator. Surg Endosc.

[6] Verhoeven DJ (2023). Assessment of minimally invasive suturing skills: is instrument tracking an accurate prediction?. J Laparoendosc Adv Surg Tech A.

[7] Langer NB, Argenziano M (2016). Minimally invasive cardiovascular surgery: incisions and approaches. Methodist Debakey Cardiovasc J.

[8] Arjomandi Rad A, Hajzamani D, & Sardari Nia P. Simulation-based training in cardiac surgery: a systematic review. Interdiscip CardioVasc Thorac Surg*. *2023. 10.1093/icvts/ivad079 PMC10435415 37220905

[9] Engelhardt S (2019). Replicated mitral valve models from real patients offer training opportunities for minimally invasive mitral valve repair. Interact Cardiovasc Thorac Surg.

[10] Dehabadi M, Fernando B, Berlingieri P (2014). The use of simulation in the acquisition of laparoscopic suturing skills. Int J Surg.

[11] Mohamed E, Elbana H (2018). Effect of simulation based training on maternity nurses’ performance and self-confidence regarding primary postpartum hemorrhage management. Am J Nurs Res.

[12] Wu S (2020). Simulation training in minimally invasive direct coronary artery bypass grafting. Heart Surg Forum.

